# A multi-layer model for the early detection of COVID-19

**DOI:** 10.1098/rsif.2021.0284

**Published:** 2021-08-04

**Authors:** Erez Shmueli, Ronen Mansuri, Matan Porcilan, Tamar Amir, Lior Yosha, Matan Yechezkel, Tal Patalon, Sharon Handelman-Gotlib, Sivan Gazit, Dan Yamin

**Affiliations:** ^1^ Department of Industrial Engineering, Tel Aviv University, Tel Aviv 69978, Israel; ^2^ MIT Media Lab, Cambridge, MA 02139-4307, USA; ^3^ Kahn Sagol Maccabi (KSM) Research and Innovation Center, Maccabi Healthcare Services, Tel Aviv, Israel; ^4^ Center for Combatting Pandemics, Tel Aviv University, Tel Aviv 6997801, Israel

**Keywords:** COVID-19, early detection, machine learning, electronic medical records

## Abstract

Current COVID-19 screening efforts mainly rely on reported symptoms and the potential exposure to infected individuals. Here, we developed a machine-learning model for COVID-19 detection that uses four layers of information: (i) sociodemographic characteristics of the individual, (ii) spatio-temporal patterns of the disease, (iii) medical condition and general health consumption of the individual and (iv) information reported by the individual during the testing episode. We evaluated our model on 140 682 members of Maccabi Health Services who were tested for COVID-19 at least once between February and October 2020. These individuals underwent, in total, 264 516 COVID-19 PCR tests, out of which 16 512 were positive. Our multi-layer model obtained an area under the curve (AUC) of 81.6% when evaluated over all the individuals in the dataset, and an AUC of 72.8% when only individuals who did not report any symptom were included. Furthermore, considering only information collected before the testing episode—i.e. before the individual had the chance to report on any symptom—our model could reach a considerably high AUC of 79.5%. Our ability to predict early on the outcomes of COVID-19 tests is pivotal for breaking transmission chains, and can be used for a more efficient testing policy.

## 1. Introduction

Severe acute respiratory syndrome coronavirus 2 (SARS-CoV-2, which causes coronavirus disease 2019, or COVID-19) was first identified in Wuhan, China, in December 2019. It has since developed into a pandemic, affecting 219 countries and territories worldwide, with over 109 million infected individuals and over 2.4 million lives lost to this deadly virus as of 18 February 2021 [1].

Despite the considerably fast development of an effective vaccine, the pandemic is expected to continue to disrupt our lives in the near future for multiple reasons. These include the emergence of highly transmissible mutant strains [2,3], the non-optimal efficacy of the developed vaccines and the current disapproval of their administration to certain populations [4], the limited supply and distribution capacities of the vaccines [5], as well as the potential risk of vaccine-waning immunity [6]. Thus, in parallel with the challenge of increasing vaccination coverage and long-term effectiveness, the implementation of early detection and prompt isolation strategies is still required in order to break transmission chains and contain local outbreaks.

Current efforts for the early detection of COVID-19 mainly rely on screening practices, which typically include a combination of reported symptoms and potential exposure to infected individuals [7]. Among the COVID-19 symptoms are loss of taste and smell, fatigue and fever, all of which have been found to be useful for the disease’s detection [7,8]. However, provided that multiple pathogens may cause symptoms similar to those of COVID-19, symptom-based detection is of limited utility. Moreover, it is inherently prone to miss presymptomatic or asymptomatic cases, which account for 40–45% of those infected with COVID-19, who can still transmit the disease [7,9]. Consequently, the USA has recently scaled up efforts to improve its testing capacity and accuracy in an unprecedented manner [10].

Several pioneering studies have offered proactive methods for COVID-19 detection based on smartwatches and activity trackers [11–13]. For example, a recent study showed that the integration of self-reported symptoms and sensor data from smartwatches resulted in an area under the curve (AUC) as high as 80% [11]. However, these methods rely on dedicated devices and require that individuals agree to frequently wear these devices and consent to share the collected information. Such devices are used by less than 20% of the population in developed countries, and are also limited to specific age groups and sub-populations. Thus, it is crucial to improve our ability to detect the disease using data that are already available regarding the entire population.

As the risk of infection is governed by individuals’ contact mixing patterns, it is crucial to account for the disease’s spatio-temporal dynamics as part of the detection task [14,15]. Furthermore, certain populations are known to be at greater risk than others of testing positive. Specifically, beyond age and gender, of great concern are the data showing the disproportionate effect of COVID-19 on ethnic and racial minorities and impoverished populations [16,17]. These populations often live in denser regions and are characterized by larger households, which puts them at greater risk of infection [18].

The risk of contracting the disease also depends on an individual’s protective behaviour, such as level of social distancing and hygiene practices. The latter correlates with the actual and perceived risks of an individual [19,20], both of which can be inferred from an individual’s medical history. Such evidence was also demonstrated in other contexts. For example, a previous study suggested that individuals who in the previous season had not yet been vaccinated against influenza and who were diagnosed with a respiratory illness were more likely to become vaccinated in the upcoming season [21]. Following the same logic, information gained from an individual’s medical history that can be linked to the actual and perceived risks may be used to predict said individual’s test results.

Here, we developed a multi-layer model for the early detection of COVID-19 infection. Our approach combines sociodemographic information about the tested individual, aggregated information on the spatio-temporal dynamics of the disease, and general information from the medical history of the individual, in addition to data collected during the testing episode. Our approach is pivotal for breaking transmission chains and can be used to substantially improve testing strategies.

## 2. Results

Our study included a random sample of 140 682 members of the Israel health maintenance organization Maccabi Healthcare Services (MHS) who were tested for COVID-19 at least once between February and October 2020. Of these individuals, 53.8% were women. The sampled individuals’ age ranged from 1 to 105 years, with a median age of 30 years (IQR: 16–49). These individuals underwent, in total, 264 516 COVID-19 tests, 16 512 (6.2%) of which were found to be positive.

Overall, we identified four layers of information that can help in predicting the outcome of a COVID-19 test: (i) the sociodemographic information of the tested individual, (ii) the spatio-temporal patterns of the disease observed around the time of the testing episode, (iii) the medical condition and general health consumption of the tested individual over the past 5 years, and (iv) the information collected on the tested individual during the testing episode.

**Figure 1.**
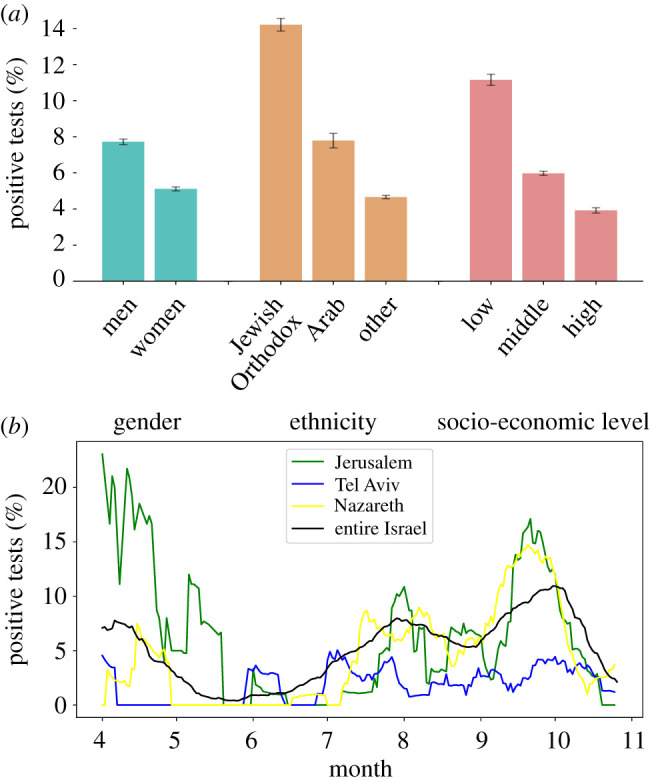
Layers 1 and 2: sociodemographic information of the tested individual and the spatio-temporal dynamics of the disease. (*a*) Percentage of positive tests stratified by gender, ethnicity, and socio-economic level. The percentages of positive tests are linked with gender, ethnicity, and socio-economic level. Error bars represent the 95% confidence interval. (*b*) Percentage of positive tests over time for three clinics located in different cities and for the entire country. The value for each day is calculated as the percentage of positive tests over the 14 days preceding this day.

In examining the sociodemographic information of the tested individuals (figure 1*a*), we found that men were more likely to test positive than women, with 7.72±0.15% positive tests for men, compared to 5.11±0.11% for women. Positive tests were also linked with ethnicity and socio-economic level. Jewish Orthodox and Arab individuals, both of whom are characterized by large households, exhibited higher percentages of positive tests (14.2±0.35% and 7.78±0.4%, respectively) than the rest of the population (4.66±0.09%). Individuals with a low socio-economic status had a substantially higher percentage of positive tests (11.15±0.31%) than those with a middle or high socio-economic status (5.97±0.12% and 3.92±0.15%, respectively). A predictive model based on this layer of information alone demonstrated a moderate classification ability between positive and negative tests, with an AUC of 67.74±0.77% (figure 3*a*).

The percentage of positive tests also varied considerably with time and across regions (figure 1*b*). Tel Aviv had lower percentages of positive tests compared to Jerusalem during most of the study period. Moreover, accounting for changes in time and region, we could identify regional outbreaks that were pivotal to our prediction task. For example, in specific zones in Nazareth, we observed lower-than-average infection rates in April but higher rates in October. Considering a predictive model based on this layer of information alone improved the ability to classify between COVID-19-positive and COVID-19-negative tests, with an AUC of 72.3±0.44% (figure 3*a*). In this analysis, we assumed that on each given day (for which we wanted to calculate the percentage of positive tests), all the relevant COVID-19 laboratory tests performed in the preceding 14 days are available (i.e. assuming a reporting lag of a single day). Examining the effect of longer lags on the model’s performance, we found that the decrease in AUC was relatively marginal. For example, for lags of 3 and 7 days, the AUC decreased by 0.75% and 2.1%, respectively (electronic supplementary material, figure S1).

Our analysis of individuals’ electronic medical records (EMRs) found that increased health consumption, increased preventative health behaviours, and certain medical conditions known to be associated with severe COVID-19 illness [22] were correlated with a lower percentage of positive tests (table 1).

**Table 1.** Layer 3: health consumption, preventative health behaviour and medical conditions. Percentages of positive tests stratified by health criterion and age group. Increased health consumption, increased preventative health behaviour, and particular medical conditions are associated with lower percentages of positive tests.

		positive tests (%) by age group							
category	feature	value	0–9	10–19	20–29	30–39	40–49	50–59	≥60
health consumption	number of hospitalizations in previous 5 years	≤2	6.34	10.4	8.48	5.66	6.06	5.69	3.43
		>2	4.39*	7.06**	6.63**	3.86**	4.23**	3.56**	2.45**
	number of visits to primary care physician in previous five years	≤5	8.07	12.31	10.49	7.15	7.35	5.99	2.26
		>5	5.98**	9.77**	7.91**	5.29**	5.75**	5.44	3.2**
	number of drug prescriptions in previous five years	≤4	7.76	11.12	9.71	6.04	6.1	5.72	4.26
		>4	4.98**	9.09**	7.37**	5.23**	5.86	5.42	3.0**
preventative behaviour	number of diagnoses in previous 5 years	≤20	8.26	12.87	10.5	7.38	6.79	5.35	2.43
		>20	5.68**	9.2**	7.65**	5.15**	5.79**	5.51	3.13*
	number of laboratory tests in previous 5 years	≤3	6.77	10.99	10.31	7.9	8.34	7.7	3.79
		>3	5.48**	9.04**	6.96**	4.63**	4.56**	4.45**	2.85**
	number of COVID-19 tests	≤1	6.23	9.65	8.97	6.44	7.55	7.64	4.74
		>1	6.81	15.05**	6.24**	2.74**	2.15**	1.87**	1.37**
	number of vaccinations in previous 5 years	0	6.62	10.83	8.81	6.2	6.46	5.74	3.15
		>0	5.75**	9.05**	7.01**	4.34**	4.9**	5.05**	3.04
medical condition	abnormal cardiovascular condition	no	6.32	10.35	8.42	5.55	5.97	5.5	3.31
		yes	3.63*	7.64*	6.67	4.53	4.66	5.4	2.63**
	abnormal blood pressure	no	6.27	10.31	8.41	5.51	6.02	5.52	3.75
		yes	0.0	8.33	6.19	6.51	5.09*	5.39	2.71**
	cancer	no	6.27	10.32	8.42	5.57	5.99	5.61	3.29
		yes	4.65	7.25	3.88*	2.54**	4.49*	3.97**	2.35**
	diabetes	no	6.27	10.32	8.39	5.51	5.94	5.39	3.04
		yes	9.09	6.31	8.53	7.54	5.76	6.31*	3.17
	chronic kidney disease	no	6.27	10.3	8.4	5.51	5.98	5.57	3.76
		yes	0.0	37.5**	7.69	7.98*	4.58*	4.71	2.4**
	chronic obstructive pulmonary disease	no	6.27	10.31	8.39	5.54	5.94	5.5	3.1
		yes	NA	0.0	0.0	5.88	3.85	4.79	2.76

Significant differences are marked with asterisks, where **denotes *p* < 0.01 and *denotes *p* < 0.05.

For example, individuals who were more likely to become vaccinated against influenza had a lower probability of testing positive across all age groups. For individuals aged 30–39, those who were vaccinated at least once in the previous 5 years received a positive result in 4.34±0.35% of the tests, whereas those who were not vaccinated at all in the previous 5 years were found to be positive in 6.2±0.31% of the tests. Likewise, individuals who were diagnosed with cancer in the past had a lower probability of testing positive, across all age groups. A predictive model based on this layer of information alone sufficed to classify between positive and negative tests, yielding an AUC of 71±0.53% (figure 3*a*).

**Figure 2.**
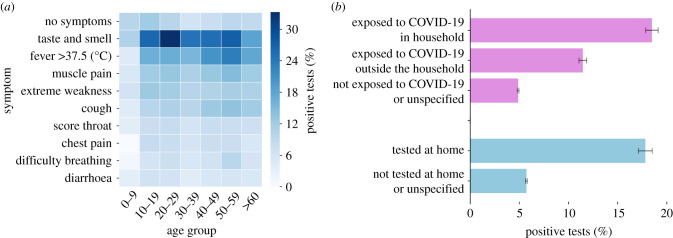
Layer 4: information collected during the testing episode. (*a*) Percentages of positive tests stratified by symptoms and age group. Several symptoms that are known to be caused by COVID-19 (e.g. loss of taste or smell) were more associated with a positive outcome. (*b*) Percentages of positive tests based on exposure to individuals with a laboratory-confirmed COVID-19 test and on the test’s location. Individuals who were exposed to infected individuals and those who were tested at home had an elevated risk of being found COVID-19 positive.

**Figure 3.**
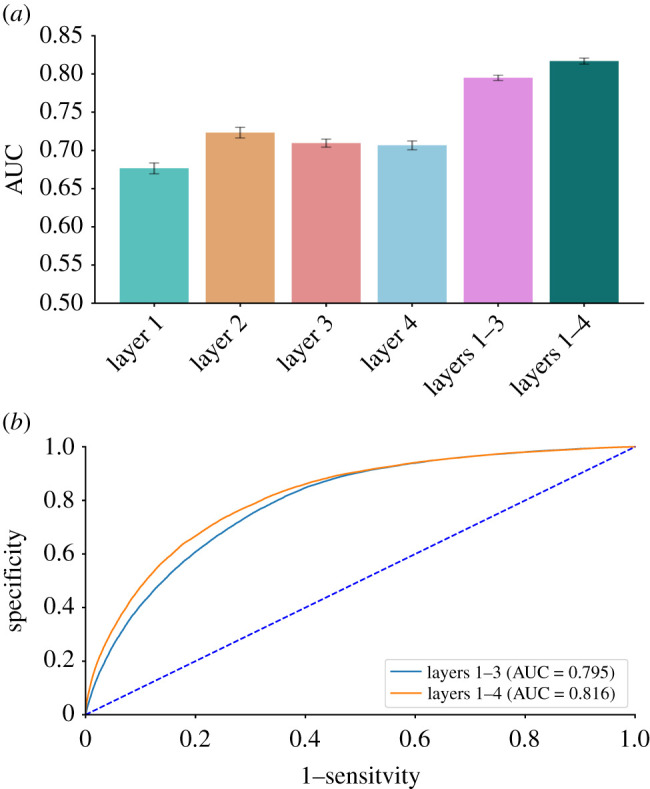
Predictive models’ performance. (*a*) Mean AUC of models based on layers 1–4 (sociodemographic information of the tested individual, spatio-temporal patterns of the disease, medical condition and general health consumption of the tested individual, and information collected during the testing episode) and the full model that combines all four layers. The full model yielded considerably better classification between COVID-19-positive and COVID-19-negative tests, with a mean AUC of 81.6%. Error bars represent the standard deviation of the 10 executions of the model. (*b*) Receiver operating characteristic curves for the full model and the model considering layers 1–3. The full model’s classification ability is only slightly better than that of the model considering the first three layers (i.e. excluding layer 4: information collected during the testing episode).

We also analysed the information collected right before the COVID-19 test was taken, during the referral and the testing episode itself. Specifically, we assessed the association between the reported symptoms and test outcome (figure 2*a*). We found that loss of taste or smell was the most indicative symptom, ranging from 10.52±0.05% of positive tests in individuals aged 0–9 to 33.16±0.03% in individuals aged 20–29. We also found that exposure to laboratory-confirmed COVID-19 individuals could serve as a predictor of test outcome. Specifically, individuals exposed to COVID-19 cases in the same household were associated with an 18.48±0.64% chance of being found positive, while individuals exposed only outside of the household were associated with an 11.45±0.39% chance of being found positive. By contrast, individuals who did not explicitly report being exposed to a known COVID-19 case had a 4.88±0.09% chance of being found positive (figure 2*b*). Moreover, we found that individuals who were tested at home had an elevated risk of being found positive (figure 2*b*). This is likely because testing at home was performed for individuals who were in quarantine or who suffered from a severe medical condition. Considering a predictive model based on this layer of information alone demonstrated the ability to classify between positive and negative tests, with an AUC of 70.6±0.59% (figure 3*a*).

A predictive model that combines all four layers of information together allowed a considerably better classification between COVID-19-positive and COVID-19-negative tests, with an AUC of 81.6±0.46% (figure 3*a*). The features derived from layers 1–3 can be collected passively prior to the testing episode. By contrast, layer 4 requires tested individuals to actively report their clinical condition. We found that a model that excludes layer 4 and uses only layers 1–3 is not only highly efficient from an operational perspective but also has a classification ability only slightly lower than that of the full model, yielding an AUC of 79.5±0.6% (figure 3*a*). This marginal difference in performance between these two models can also be observed in figure 3*b*, which presents their full receiver operating characteristic curves.

With the exception of the location of the testing, all the considered features can be collected and assessed remotely via phone calls or digital questionnaires. Thus, we also considered a model that excludes the feature indicating the testing location. We found this model to be highly informative, with its AUC score reaching 81.45±0.49%. Lastly, limiting our predictions only to individuals who did not report any symptom, our full model yielded an AUC of 72.8±0.85%. This finding demonstrates a moderate, yet considerable, ability to identify presymptomatic or asymptomatic individuals.

## 3. Discussion

We found that when using multiple layers of information, the risk of testing positive for COVID-19 is highly predictable, with the AUC reaching 81.6%. Specifically, we identified four layers of information that can predict positive COVID-19 test outcomes: (i) the sociodemographic characteristics of the tested individual, (ii) the spatio-temporal patterns of the disease observed around the time of the testing episode, (iii) the medical condition and general health consumption of the tested individual over the past 5 years, and (iv) the information reported by the tested individual during the testing episode.

We found that by relying on information from the testing episode alone (e.g. symptom-related questions), we could achieve an AUC of 70.6%. This result is consistent (albeit lower) with recent studies that showed AUCs of 72% [11] and 76% [7]. When we considered only the information collected before the testing episode—that is, before the individual had the chance to report on any symptoms—our model could reach a considerably higher AUC, 79.5%. This finding is pivotal for earlier detection. The marginal difference in AUC scores between the full model and the model without the testing episode information suggests that most of the information gained from the testing episode can be inferred from the individual’s medical history, as well as other aggregated information with regard to the disease dynamics. Moreover, while symptom-based predictions are likely to be sensitive to COVID-19 variants and the emergence of other respiratory infections, our approach is likely to be more robust, as it explicitly considers the spatio-temporal dynamics of COVID-19.

We found that individuals with underlying medical conditions and individuals who maintain a more preventative lifestyle are at lower risk of testing positive for COVID-19. This finding implies that those populations tend to better protect themselves against the disease or are more likely to be tested. While we cannot separate between the two causes, health behaviour models, including the Health Belief Model [23], and social cognitive theory [24,25] suggest that the combination of these causes is likely. Despite the inherent differences in the perception of risk between cultures worldwide, we believe that the behavioural patterns and the predictive models we have developed can be reproduced with minor adaptations in most developed countries.

For privacy purposes, we considered only general information from EMRs to infer an individual’s health condition and preventative behaviour. For example, in our model, we included information about the total number of yearly visits to one’s primary care physician and the number of medications prescribed to a patient rather than more invasive information, such as the type of prescribed medication. Clearly, more detailed information about individuals may provide an improved understanding of their behaviour and lead to improved predictive models. However, this comes at the price of invading privacy, an issue that is no less important [26].

In this study, we analysed a random sample of 140 682 individuals who were tested for COVID-19. While our approach may aid decision-makers in a postmortem fashion, namely, after a decision to take a test has been made, findings should not be extrapolated to the general population. Specifically, by accounting for the predicted probability of a test to be found positive, our work can help prioritize the order of test samples, optimize pool testing, or even recommend quarantine for specific individuals until their test results arrive. By contrast, for policy-related questions concerning the general population, including targeted screening or identifying asymptomatic individuals in the community, a proper control trial is essential.

Our study identifies and relies on correlations and associations in both pattern analysis and predictive modelling and does not attempt to assume or imply causality. In addition, this study does not explicitly account for attempted interventions by MHS during the study period, including efforts to test individuals at higher risk. Moreover, the sensitivity and specificity of RT-PCR testing vary considerably among different age groups and among individuals with different levels of infection severity or at different stages of disease progression [27]. Specifically, the sensitivity in mild cases could be as low as 62.5% [7,28], and the sensitivity a day prior to symptom onset falls below 33%.

Our methodological approach which considers these four different layers of information is likely applicable to other infectious diseases besides COVID-19. Specifically, in settings of airborne infections such as measles or pertussis, both the basic reproductive number and the vaccination coverage in the general population are relatively high. Thus, we typically observe pockets of outbreaks in regions where vaccine refusal is higher [29] and in subpopulations with high fertility, in which a large proportion of infants is yet to be vaccinated [30,31]. Additionally, several airborne diseases, as opposed to others, are characterized by periodicity [32,33]. Accordingly, we expect that spatio-temporal information (layer 1) and socio-economic information (layer 2) will be valuable for detection. When it comes to sexually transmitted diseases, multiple factors, including sexual orientation, unprotected intercourse exposures and the number of sex partners, may remain relatively invariable over time [34]. Therefore, individuals tested, diagnosed or treated in the past are at higher risk of being found positive [34], making behavioural information (layer 3) valuable for detection.

In conclusion, COVID-19 test results are highly predictable and can be achieved even in the absence of detailed information on the signs and symptoms of the individual during the testing episode. The ability to predict the outcomes of COVID-19 tests in real time can be used to formulate a more efficient testing policy. In the post-vaccine era, such a policy may become even more efficient due to lower transmission rates, enabling easier differentiation between positive and negative COVID-19 tests.

## 4. Methods

### 4.1. Ethical considerations

The study was approved by MHS’ Helsinki institutional review board, protocol number 0093-20-MHS, signed on 21 October 2020. Informed consent was waived as identifying details were removed before the analysis.

### 4.2. Study population and case definition

We analysed the anonymized EMRs of 140 682 randomly sampled individuals tested at least once with PCR for COVID-19 during February–October 2020. The individuals were members of MHS. MHS is the second largest health maintenance organization in Israel, serving more than 25% of the Israeli population (approx. 2.5 million members). MHS members are representative of the Israeli population and reflect all demographic, ethnic and socio-economic groups and levels [35].

For the 140 682 individuals considered in this study, 279 140 COVID-19 tests were performed during the examined time period. According to previous guidelines in Israel, individuals who tested positive were motivated to conduct additional tests to terminate self-quarantine. Since our goal was to predict the presence of COVID-19, for each individual, we included in our analysis only tests until his/her first positive test (if such existed), which corresponded to 264 517 tests in total.

For each individual, we extracted data from their EMRs between 2015 and 2020. Specifically, we compiled four layers of information to predict COVID-19 test outcomes: (i) the sociodemographic information of the tested individual, (ii) the spatio-temporal patterns of the disease, (iii) the medical condition and general health consumption behaviour of the tested individual and (iv) the information collected from the tested individual during the test procedure. Information on features considered for each of the layers is detailed in electronic supplementary material, table S4.

### 4.3. Statistical analysis

To examine the statistical significance between the proportions of positive tests for two different groups (e.g. diabetic individuals versus non-diabetic individuals, table 1), we used the two proportions Z-test. In settings for which the conditions to perform a Z-test were not satisfied, we compared the proportions assuming a beta distribution for each proportion, with parameters *α* and *β* representing the number of positive cases + 1 and the number of negative cases + 1, respectively. To compare statistical differences between more than two groups, we used *χ*^2^ test of independence. The problem of determining the outcome of a COVID-19 test (i.e. positive or negative) was treated as a machine learning, binary classification task. Specifically, we generated six different prediction models, based on single layers of information (sociodemographic, spatio-temporal, health-related and test-related), as well as on combination of layers (before the test, and before and during the test).

For our models, we considered the following classification algorithms: (i) XGBoost [36], (ii) Naïve Bayes, (iii) logistic regression and (iv) artificial neural network. In the main text, we report the results of the XGBoost classifier as it yielded the best classification performance. Figure S2 in the electronic supplementary material reports our experimentation with the three other classification algorithms. In all cases, we used default parameters, after confirming that applying hyperparameters tuning over the training set using grid search afforded comparable results.

We evaluated the model using a 10-fold cross-validation process, where each time, the model was trained using 90% of the data and then tested over the remaining 10%. We chose *k* = 10 since our dataset is relatively large, and the observation that this value has been shown empirically to yield test error rate estimates that suffer neither from excessively high bias nor from very high variance in general [37], and since it was previously used in settings similar to ours [38]. The reported results are the mean of these 10 executions. The area under the receiver operating characteristic curve (AUC) was used as the main metric to assess the overall performance of the trained models.

## Data accessibility

Access to the data used for this study can be made available upon request and is subject to internal review approval from the institutional review board of MHS with the current data sharing guidelines of MHS and Israeli law.
